# Exploring the relationship between the Mediterranean diet and weight loss maintenance: the MedWeight study

**DOI:** 10.1017/S0007114520001798

**Published:** 2020-05-21

**Authors:** Dimitrios Poulimeneas, Costas A. Anastasiou, Inês Santos, James O. Hill, Demosthenes B. Panagiotakos, Mary Yannakoulia

**Affiliations:** 1 Department of Nutrition and Dietetics, School of Health Sciences & Education, Harokopio University, GR 17671 Athens, Greece; 2 Centro Interdisciplinar de Estudo da Performance Humana, Faculty of Human Kinetics, University of Lisbon, 1499-002 Lisbon, Portugal; 3 Laboratory of Nutrition, Faculty of Medicine, University of Lisbon, 1649-028 Lisbon, Portugal; 4 Department of Nutrition Sciences, University of Alabama at Birmingham, Birmingham, AL 35233, USA

**Keywords:** Dietary patterns, Mediterranean diet, Obesity, Weight control, Weight regain

## Abstract

Weight loss maintenance is crucial for obesity management, yet optimal dietary patterns for this period are not established. We aimed to explore the relationship between adherence to the Mediterranean diet and weight loss maintenance. Sample includes 565 adults (62 % women) of the MedWeight study. Eligible volunteers were those reporting intentional weight loss of ≥10 %, starting from a BMI ≥ 25 kg/m^2^, over 12 months prior to enrolment. Based on current weight, participants were characterised as maintainers (≤90 % maximum weight) or regainers (>95 % maximum weight). Socio-demographics and weight history were recorded. Dietary intake was assessed by two non-consecutive 24-h recalls within 10 d and analysed in energy, macronutrient and food group intakes. Adherence to the Mediterranean diet was assessed with the Mediterranean Diet Score (MedDietScore) (range 0–55, greater scores showing higher adherence). Protein intake was higher in maintainers than in regainers (*P* < 0·001). When MedDietScore quartiles were considered, a linear trend for weight loss maintenance was revealed (*P* < 0·05). After adjustment for basic demographic characteristics, being in the third or fourth quartile of the MedDietScore (*v.* first) was associated with 2·30 (95 % CI 1·29, 4·09) and 1·88 (95% CI 1·10, 3·22) increased odds of maintenance. Regarding individual MedDietScore components, only fruit intake is associated with increased odds for maintenance (1·03 (95% CI 1·01, 1·06)). The leave-one-out approach revealed that at least six MedDietScore components were essential for the observed relationship. Higher adherence to the Mediterranean diet was associated with 2-fold increased likelihood of weight loss maintenance. Future studies should replicate these findings in non-Mediterranean populations as well.

The term Mediterranean diet is used to describe the traditional dietary pattern of the people residing in the Mediterranean basin, characterised by abundance of plant foods such as fruits, vegetables, either as main or side dish, cereals, including bread, legumes, nuts and seeds. Olive oil is the principal source of fat. The Mediterranean diet also includes moderate amounts of dairy products, low to moderate amounts of fish and poultry, red meat in low amounts and wine, consumed modestly, mostly with meals^(1,2)^. Strong evidence supports that higher adherence to this traditional dietary pattern is associated with greater longevity^(3)^ and acts both as a preventive and therapeutic target for many prevailing non-communicable diseases^(4-6)^.

The Mediterranean diet has also been utilised as a whole-diet approach model within the framework of obesity prevention and management. Higher adherence to the Mediterranean diet has been found to be associated with lower body weight^(7)^, to prevent long-term weight gain^(8-10)^ and to produce significant weight loss, with or without energy restriction^(11)^. Nevertheless, much less is known regarding its relationship with long-term weight loss maintenance.

Although weight loss maintenance is considered an integral part of obesity management, optimal dietary patterns for successful weight loss maintenance have not been established^(12)^. Few studies have evaluated different diets during the maintenance of weight loss. We have previously observed that an *a posteriori*-defined healthy dietary pattern was significantly associated with weight loss maintenance^(13)^. Similar results have been found for high-protein diets, with or without alterations in glycaemic index^(14,15)^. Two interventional weight loss studies with 6 months of follow-up suggest that adhering to the Mediterranean diet after weight loss may favour maintenance outcomes^(16,17)^. However, to the best of our knowledge, no study to date has examined the effects of the Mediterranean diet on maintaining weight loss for prolonged periods of time (i.e. more than 12 months). Our hypothesis was that higher adherence to the Mediterranean dietary pattern in the post-dieting period is associated with favourable outcomes for the maintenance of reduced body weight. To address this hypothesis, we explored the relationship between adherence to the Mediterranean diet and long-term weight loss maintenance in the MedWeight study^(18)^, a large cohort of weight loss maintainers and regainers, reporting lifestyle data for at least 12 months after initial weight loss.

## Methods

### Study design and population

The MedWeight study is an ongoing Greek registry of individuals with a history of overweight or obesity, residing in Greece. The detailed study protocol has been published elsewhere^(18)^. In short, inclusion criteria were (i) age 18–65 years, (ii) a lifetime maximum BMI ≥ 25 kg/m^2^ and (iii) intentional weight loss of at least 10 % of maximum weight, in the period >12 months prior to enrolment. Currently, or within the previous year, pregnant women were excluded from sampling. Participation in the study was conducted through the study’s website (medweight.hua.gr). According to their current weight, the study’s algorithm automatically classified participants as maintainers, for attaining current weight ≤90 % of their maximum weight, or regainers, for attaining current weight >95 % of their maximum weight. To avoid overlap between groups, individuals with a weight of 90–95 % of their maximum weight were excluded from the study. This decision was based on (i) the weight loss maintenance definition by Wing & Hill^(19)^, suggesting that successful maintainers are those who maintain a loss of at least 10 % of their maximum weight for over 12 months and (ii) ascertaining that regainers were attaining a current body weight below the clinically meaningful weight loss range of 5–10 ^(20)^. Then, eligible participants were asked to report on their current characteristics and habits (those at the time of recruitment). For the present analysis, 565 volunteers were analysed (69·7 % maintainers and 62·1 % women). The Ethics Committee of Harokopio University, Greece, approved the study protocol, and all participants provided electronic informed consent.

### Demographics characteristics

Age (in years), sex (man/woman), education level that was assessed by years of formal education and family status categorised as (a) single, (b) married or cohabitating, (c) divorced or (d) widowed and afterwards coded as married or else (due to the small number of participants in categories c and d) were recorded.

### Assessment of weight history and physical activity

Specific weight history questions were asked to the participants, regarding current anthropometric characteristics (weight (in kg) and height (in m), from which BMI was computed as weight/height^2^ (kg/m^2^)), maximum weight ever reached and initial weight loss achieved (as percentage of maximum weight). For maintainers, additional questions regarding time maintaining weight loss and the amount of weight loss they are currently maintaining (as percentage of maximum weight) were computed.

Participants’ activity levels were assessed through the validated Greek short version of the International Physical Activity Questionnaire^(21)^. Volunteers reported how much time they spent during the previous week for vigorous and moderate activities and walking. This allowed for calculating total metabolic equivalent of task minutes (MET-minutes) of activity during the previous week, which were then converted to energy expenditure (kJ/d from physical activity), using the MET-minutes × weight/60 equation^(22)^

### Dietary intake assessment

Trained researchers conducted two non-consecutive telephone 24-h recall dietary recalls, based on the multiple-pass method^(13,23)^. All participants were asked for all food and beverages they consumed during the previous day. The same researcher conducted both dietary recalls within 10 d, with weekdays and weekends proportionally represented among participants. Researchers were blind to the maintenance status (maintainer or regainer) of the volunteers.

Recall data were analysed in terms of energy intake and macronutrients, using relevant software (Nutritionist Pro 2007; Axxya Systems). For Greek foods not available in the software databases, recipes were broken down to their original ingredients, and/ or the food label of the actual product was crosschecked with the nutrient analysis of the selected food in the database. Recall data were also analysed in terms of food group consumption^(13)^, and intake of all major food groups was estimated, including the core foods of the Mediterranean diet. Food group consumption was expressed as servings/week^(24)^, with the exception of olive oil which was expressed as frequency of using olive oil in meals.

### Adherence to the Mediterranean diet

Adherence to the Mediterranean diet was assessed by the Mediterranean Diet Score (MedDietScore) proposed by Panagiotakos *et al.*^(25)^. The scoring is based on the weekly consumption of eleven food groups. For non-refined cereals, fruits, vegetables, legumes, potatoes, fish and olive oil, individuals who reported no consumption were assigned a score of 0, and scores of 1–5 are assigned for rare to daily consumption. For meat and meat products, poultry and full-fat dairy products, scores were assigned on a reverse scale. For alcohol intake, it is assumed that small amounts of consumption are beneficial, while high or zero consumption is detrimental. Thus, a score of 5 was assigned for consumption of <300 ml of alcohol/d and more than zero, a score of 0 was assigned for no consumption or for consumption of 700 ml/d or more and scores of 4–1 were assigned for consumption of 600–700, 500–600, 400–500 and 300–400 ml/d (100 ml has 12 g of ethanol concentration), respectively. The total score ranges from 0 to 55, with higher values indicating greater adherence to the Mediterranean dietary pattern.

### Statistics

Data distribution was graphically explored with Q–Q plots in order to assess normality. Normally distributed continuous variables were presented as means and standard deviations, and non-normally distributed variables as medians and quartiles (Ql, Q3). Independent-sample *t* tests and Mann–Whitney *U* tests for (normally and non-normally distributed, respectively) continuous variables and *χ*^2^ tests for categorical variables were used to examine differences between maintainers and regainers. To further explore the relationship between weight loss maintenance and adherence to the Mediterranean diet, quartiles of the MedDietScore were calculated (≤21, 22–25, 26–30, ≥31); the association between adherence to the MedDietScore quartiles and maintenance status (i.e. maintainers or regainers) was examined applying multi-adjusted logistic regression (results are presented as OR and 95 % CI). Three models were employed, model 1: crude/unadjusted; model 2: adjusted for sex, age, marital status and years of education; model 3: adjusted for sex, age, marital status, years of education and energy intake and physical activity. We also performed additional adjustment for the variables described in model 3, for smoking habits (current or non-smoker) and sleep habits (mean duration of nocturnal sleep during the last month). Addition of variables in the nested models was based on their theoretically hypothesised association with the outcome. The Hosmer–Lemeshow test was used to evaluate models’ goodness of fit. Similarly, multi-adjusted logistic regression models were used to explore the relationship between the core food groups of the Mediterranean diet and weight loss maintenance. Additionally, in order to cross-validate the role of each component of the MedDietScore on weight loss maintenance, analyses based on the leave-one-out approach were conducted. In this regard, the MedDietScore was recalculated with ten instead of eleven items, and eleven new multi-adjusted models were estimated. Statistical analyses were performed using STATA version 15 software (M. Psarros & Associates).

## Results

Participants’ general characteristics, according to maintenance status, are presented in Table 1. Maintainers exhibited maintenance of a 22·3 (sd 9·4)% weight loss for a median period of 2·8 (Q1 1·7, Q3 5·7) years. Compared with regainers, maintainers were younger and less frequently married, had a lower current BMI and a higher initial weight loss (as percentage of maximum weight) (*P* < 0·001 for all comparisons). Moreover, maintainers were found to be more active than regainers, reporting greater daily activity energy expenditure from physical activity (1552 (Q1 699, Q3 3017) *v.* 1109 (Q1 515, Q3 2272) kJ/d, *P* = 0·006). Current smoking was not different between groups (maintainers 36·6%; regainers 27·0%, *P* = 0·096). Maintainers reported greater nocturnal sleep duration than regainers (7·0 (sd 1·2) *v.* 6·7 (sd 1·3)h, *P* = 0·022).

Comparisons of dietary intake by maintenance status are presented in Table 2. On average, maintainers consumed more protein compared with regainers (0·98 (Q1 0·74, Q3 1·28) *v.* 0·82 (Q1 0·60, Q3 1·05) g/kg body weight, *P* < 0·001). Maintainers had marginally significantly higher consumption of fruit compared with regainers (8·8 (sd 9·1) *v.* 7·3 (sd 8·1) servings/week, *P* = 0·06). No other significant differences in the consumption of the core food groups of the Mediterranean diet, or in the total score of adherence to the Mediterranean diet, were found between maintainers and regainers.

However, residual confounding may exist, and the aforementioned results from the crude analyses may be prone to bias due to sampling alterations. The estimated multi-adjusted logistic regression models showed a linear trend between quartiles of the MedDietScore and weight loss maintenance (*P* < 0·05, see Table 3). After adjustment for basic demographic characteristics (sex, age, years of education and marital status), being in the third or the fourth quartile of the MedDietScore (*v.* in the 1st) was associated with 2·30 (95 % CI 1·29, 4·09) and 1·88 (95 % CI 1·10, 3·22) increased odds for being a maintainer than a regainer, respectively. These findings remained significant after additional adjustment for energy intake and physical activity. Further adjustment for smoking habits and sleep habits did not affect statistically significant findings.

No statistically significant results emerged from the multi-adjusted logistic regression models examining the relationship between food group consumption and weight loss maintenance, apart from the fruits group (Table 4). Specifically, in the adjusted and fully adjusted models (for the confounders mentioned above), a significant trend for being a maintainer as compared with regainer towards increased consumption of weekly servings of fruit was revealed (OR/one fruit serving per week 1·03 (95 % CI 1·01, 1·06)); that is, every weekly serving of fruit increase was associated with 3% greater odds of being a maintainer.

The leave-one-out approach showed that the MedDietScore was not associated with weight loss maintenance in six of the eleven altered MedDietScore models. These were the scores that were calculated without the respective loadings from potatoes (OR 1·62 (95 % CI 0·91, 2·90)) without the respective loadings from potatoes, fruit (OR 1·70 (95 % CI 0·94, 3·05)), legumes (OR 1·38 (95 % CI 0·77, 2·47)), olive oil (OR 1·66 (95 % CI 0·94, 2·94)), red meat (OR 1·74 (95 % CI 0·99, 3·06)) and alcohol (OR 1·52 (95 % CI 0·88, 2·62)) weekly consumption.

## Discussion

This is one of the first studies exploring the relationship between adherence to the Mediterranean diet and long-term weight loss maintenance. Our results suggest that high adherence to the Mediterranean diet is associated with a 2-fold increased likelihood of weight loss maintenance in this large cohort of maintainers and regainers. This favourable association was mainly attributed to the Mediterranean diet as a whole dietary pattern. However, some components of this traditional pattern may largely contribute to the observed association.

Although mean adherence to the Mediterranean diet did not differ between maintainers and regainers, a linear trend for weight loss maintenance was revealed in the unadjusted model. After adjustment for potential confounding factors, our results highlighted that higher adherence to the Mediterranean diet confers benefits for long-term weight loss maintenance. Indeed, a large body of evidence supports our finding, given that scoring high (against low) in Mediterranean diet indexes is associated with favourable health and weight outcomes (i.e. better fasting glucose homeostasis indices^(26)^ and lipid profile^(27)^, less longitudinal weight gain^(28)^ and lower likelihood of obesity^(29)^).

In the PREDIMED-Plus trial, a 6-month weight loss intervention based on energy-restricted Mediterranean diet, the intervention group continued to lose weight and enhance their adherence to the pattern from intervention end to the 12-month follow-up phase^(16)^. In addition, combination of a biphasic very low-energy diet (40 d) with two periods of maintenance (4 and 6 months) based on the Mediterranean diet has been associated with a reduction of about 15 kg and null weight regain in participants with obesity, over a 12-month period^(17)^. Thus, findings of these interventions, along with our results, support the beneficial role of the high adherence to the Mediterranean diet during the post-dieting period. There are several physiological mechanisms by which the Mediterranean diet may exert significant benefits for weight loss maintenance^(30)^. Specifically, this dietary pattern provides a large quantity of dietary fibre, which increases satiety and satiation through prolonged mastication, increased gastric detention and enhanced release of cholecystokinin; has a low energy density and a low glycaemic load, potentially leading to better appetite control; and has high water content, which taken together with the previously mentioned characteristics contribute to increased satiation and consequently to lower energy intake, thus promoting the maintenance of weight loss^(30)^.

Our results also support the beneficial role of protein intake in weight loss maintenance, as previously reported in other weight control registries^(31)^. While results from interventional studies remain controversial^(32)^, some studies suggest that modest increments in protein intake in the post-dieting period are associated with better maintenance of the lost weight, possibly through the effects of higher protein effect in satiety and increased energy expenditure^(15,33)^.

Furthermore, our results showed that the consumption of fruits, which are rich in dietary fibre and water and thus have low energy density, was positively associated with weight loss maintenance. Previous evidence demonstrated that increased intake of fruit was inversely associated with longitudinal weight gain^(34,35)^. Additionally, in the Weight Loss Maintenance Trial, a multi-centre, randomised controlled trial on the 6-month weight loss intervention followed by a 30-month maintenance phase, every increase in the serving of fruit in the maintenance phase is associated with 0–04 kg of weight loss^(36)^. The potential underlying mechanisms are largely unknown. A meta-analysis concluded that increased consumption of fruit and vegetables is unlikely to result in weight gain and may even produce modest long-term weight reduction^(37)^. We postulate that the observed relationship may reflect a possible substitution of high-energy snacks with fruit among maintainers, as a weight control behaviour. On the other hand, even though specific food groups may withhold promising aspects for weight loss maintenance, people do not consume foods in isolation. Decomposing a dietary pattern to its components may undermine the interactive or synergistic effects of foods that are consumed in combinations in real life^(38)^.

Consequently, analyses on the relationship between various combinations of food groups and weight loss maintenance highlighted the possibility that other food groups, in addition to fruit, may be of importance. In specific, the leave-one-out approach highlighted that higher consumption of not only fruit but also potatoes, legumes and olive oil, modest consumption of alcohol and lower consumption of red meat and products may be important for weight loss maintenance. Taken together, these results are supportive of the synergistic effect of the Mediterranean diet’s components^(39,40)^ on weight loss maintenance, rather of the observed relationship being dependent on individual effects of single dietary constituents^(29)^. Similar results were also observed in the PREDIMED-Plus trial, where the intervention group reported greater adherence to the guidance regarding the Mediterranean diet’s components of fruits, vegetables, legumes, commercial bakery, sauces, added sugars, red meat and poultry than controls during the maintenance phase^(16)^.

The present study has several strengths. Findings reported here are from the largest European weight control registry. The inclusion of regainers in the sample allowed for direct comparisons of the measures acquired across long-term weight loss outcome groups. Dietary intake data were collected through thorough dietary assessment, by means that have been proven adequate for assessing nutrient intake on a population basis^(41)^. The cross-sectional nature of the present study reveals association, but not causality. On the other hand, we believe that our findings provide the basis for future experimental studies, given that performing trials for all possible prudent diets may not be entirely pragmatic or feasible. Although we performed various adjustments, other confounders, not taken under consideration by the present study, may have had an impact on the observed relationship (e.g. presence of co-morbidities or stress). We examined the adoption of the Mediterranean diet in a Mediterranean cohort, which may limit the generalisation of our results to other populations. Nevertheless, this dietary pattern can be transferable^(42,43)^ and several studies showed that it has produced significant health outcomes to Mediterranean and non-Mediterranean populations alike^(44,45)^.

In conclusion, higher adherence to the Mediterranean diet was associated with a 2-fold increased likelihood of weight loss maintenance. Our results highlight the potential beneficial effect of this plant-based dietary pattern in long-term obesity management, as well as provide novel targets for diet planning during weight loss maintenance. Future research should explore the effects of the adoption of a Mediterranean dietary pattern in the post-dieting period through adequately designed interventions with long-term follow-up periods, as well as test the level of transferability of these findings to non-Mediterranean populations.

## Figures and Tables

**Table 1.** Participants’ general characteristics, by maintenance status (*n* 565) (Mean values and standard deviations; medians and quartiles; relative frequencies in percentages)

	Maintainers (*n* 394)				Regainers (*n* 171)			
	Mean	%		SD	Mean	%		SD	*P*
Sex									
Women		61·2				64·3			0·477
Age (years)	31·8			9·9	36·3			10·7	<0·001
Years of education	15·7			3·4	15·7			3·3	0·957
Employed		86·6				84·2			0·476
Married		23·8				47·5			<0·001
Current weight (kg)	75·6			15·2	92·2			19·1	<0·001
Current BMI (kg/m^2^)	25·7			4·3	31·5			5·2	<0·001
Maximum weight (kg)	99·0			25·1	95·5			19·7	0·005
Max BMI (kg/m^2^)	33·6			7·5	32·7			5·3	0·627
Initial weight loss	26·8			9·7	18·5			7·0	<0·001
(% maximum weight)									
Maintained weight loss (% maximum weight)	22·3			9·4	-				-
Maintenance duration (years)									
Median			2·8				-		
Q1,Q3			1·7, 5·7				-		

**Table 2.** Dietary intake and consumption of the core food groups of the Mediterranean diet, by maintenance status (*n* 565). (Mean values and standard deviations; medians and quartiles)

	Maintainers (*n* 394)				Regainers (*n* 171)			
	Median	Mean	sd	Quartiles	Median	Mean	sd	Quartiles	*P*
Energy intake (kJ/d)	6987			5586, 8745	7314			5724, 9021	0·626
Protein (% total daily EI)	16·8			14·4, 19·9	16·6			14·3, 19·7	0·585
Protein (g/kg BW)	0·98			0·74, 1·28	0·82			0·60, 1·05	<0·001
Carbohydrates (% total daily EI)		43·4	9·6			43·4	8·5		0·954
Lipids (% total daily EI)		38·3	8·9			38·8	8·5		0·529
Alcohol (% total daily EI)	0·0			0·0, 3·8	0·0			0·0, 2·8	0·370
Fibre (g/d)	15·0			10·7, 22·2	14·7			10·5, 21·5	0·483
Food group consumption (servings/week, with the exception of olive oil consumption, which is presented as frequency of using olive oil in meals)								
Non-refined cereal		10·5	12·2			9·4	10·0		0·301
Potatoes	0·0			0·0, 5·9	0·0			0·0, 7·0	0·794
Fruit		8·8	9·1			7·3	8·1		0·063
Vegetables		14·8	12·7			15·2	11·1		0·747
Legumes	0·0			0·0, 1·4	0·0			0·0, 0·0	0·511
Fish	0·0			0·0, 0·0	0·0			0·0, 1·4	0·617
Olive oil		7·8	4·3			7·9	4·2		0·764
Red meat and products		8·1	8·9			9·2	8·7		0·171
Poultry		4·7	6·8			4·1	6·3		0·282
Full-fat dairy products		10·7	10·5			11·0	11·1		0·761
Alcohol	0·0			0·0, 3·5				0·0, 3·5	0·283
MedDietScore (0–55)		26·6	6·8			25·7	6·6		0·157

EI, energy intake; BW, body weight; MedDietScore, Mediterranean Diet Score.

**Table 3.** Logistic regression models describing the relationship between adherence to the Mediterranean diet and weight loss maintenance (Odds ratios and 95 % confidence intervals)

MedDietScore (0–55)	Q1 (*n* 148)		Q2 (*n* 130)		Q3 (*n* 135)		Q4 (*n* 152)		*P*_for trend_
	≤21		22–25		26–30		≥31		
	OR	95% CI	OR	95% CI	OR	95% CI	OR	95% CI	
Unadjusted	1·00	Reference	1·09	0·67, 1·79	1·66	0·99, 2·77	1·56	0·95, 2·56	0·031
Adjusted*	1·00	Reference	1·29	0·76, 2·22	2·30	1·29, 4·09	1·88	1·10, 3·22	0·006
Fully adjusted†	1·00	Reference	1·31	0·76, 2·27	2·29	1·28, 4·11	1·80	1·04, 3·12	0·006

MedDietScore, Mediterranean Diet Score.

* Adjusted for sex, age, years of education and marital status (married or not).

† Adjusted for sex, age, years of education, marital status (married or not), energy intake (kJ/d) and physical activity energy expenditure (kJ/d).

**Table 4.** Logistic regression models describing the relationship between the core food groups of the Mediterranean diet and weight loss maintenance (Odds ratios and 95 % confidence intervals)

Food group consumption (servings/week)	Unadjusted model		Adjusted model*		Fully adjusted model†	
	Weight loss maintenance					
	OR	95 % CI	OR	95% CI	OR	95% CI
Non-refined cereal	1·01	0·99, 1·03	1·01	0·99, 1·03	1·01	0·99, 1·03
Potatoes	0·98	0·95, 1·01	0·97	0·95, 1·00	0·98	0·94, 1·01
Fruit	1·02	0·99, 1·04	1·03	1·01, 1·06	1·03	1·01, 1·06
Vegetables	0·99	0·98, 1·01	0·99	0·98, 1·01	0·99	0·98, 1·02
Legumes	1·02	0·99, 1·05	1·02	0·98, 1·05	1·01	0·98, 1·05
Fish	0·99	0·95, 1·03	0·98	0·94, 1·02	0·98	0·94, 1·02
Red meat and products	0·99	0·97, 1·01	0·99	0·97, 1·01	0·99	0·97, 1·02
Poultry	1·02	0·99, 1·04	0·99	0·96, 1·03	0·99	0·97, 1·03
Full-fat dairy products	0·99	0·98, 1·01	1·00	0·98, 1·02	1·01	0·99, 1·03
Alcohol	1·00	0·97, 1·03	0·99	0·97, 1·03	1·00	0·97, 1·03

* Adjusted for sex, age, years of education and marital status (married or not).

† Adjusted for sex, age, years of education, marital status (married or not), energy intake (kJ/d) and physical activity energy expenditure (kJ/d).

## Abbreviation:

MedDietScore: Mediterranean Diet Score
